# Delta-T Flicker Noise Demonstrated with Molecular Junctions

**DOI:** 10.1021/acs.nanolett.3c04445

**Published:** 2024-01-31

**Authors:** Ofir Shein-Lumbroso, Matthew Gerry, Abhay Shastry, Ayelet Vilan, Dvira Segal, Oren Tal

**Affiliations:** † Department of Chemical and Biological Physics, Weizmann Institute of Science, Rehovot 7610001, Israel; ‡ Department of Physics, University of Toronto, 60 Saint George Street, Toronto, Ontario M5S 1A7, Canada; § Department of Chemistry and Centre for Quantum Information and Quantum Control, University of Toronto, 80 Saint George Street, Toronto, Ontario M5S 3H6, Canada

**Keywords:** flicker noise, 1/*f* noise, molecular junction, atomic contact, thermal noise, quantum transport

## Abstract

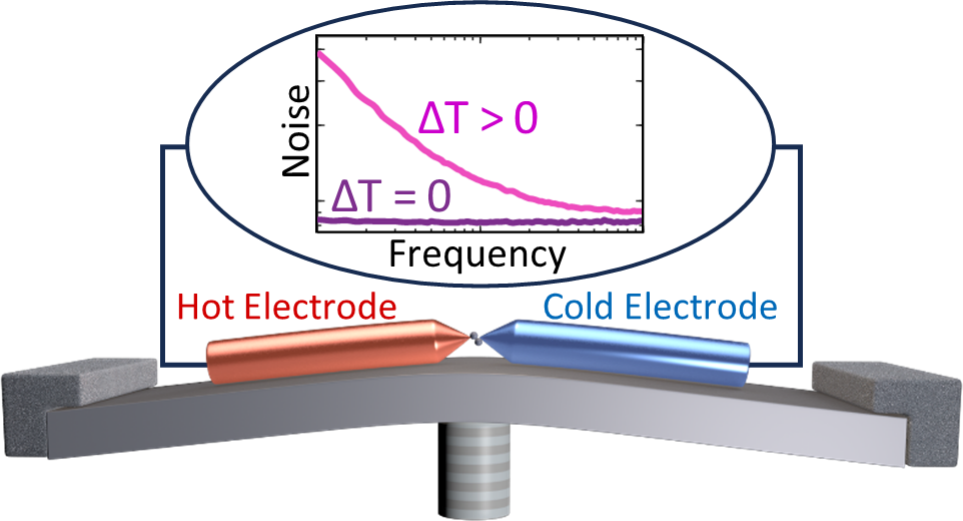

Electronic flicker noise is recognized as the most abundant noise in electronic conductors, either as an unwanted contribution or as a source of information on electron transport mechanisms and material properties. This noise is typically observed when a voltage difference is applied across a conductor or current is flowing through it. Here, we identify an unknown type of electronic flicker noise that is found when a temperature difference is applied across a nanoscale conductor in the absence of a net charge current or voltage bias. The revealed delta-T flicker noise is demonstrated in molecular junctions and characterized using quantum transport theory. This noise is expected to arise in nanoscale electronic conductors subjected to unintentional temperature gradients, where it can be a performance-limiting factor. On the positive side, delta-T flicker noise can detect temperature differences across a large variety of nanoscale conductors, down to atomic-scale junctions with no special setup requirements.

Flicker noise, sometimes known as 1/*f* noise due to its inverse dependence on frequency, is a ubiquitous phenomenon in nature.^1−8^ The electronic version of flicker noise can be found in most electronic conductors or devices,^1,2,6,7,9−17^ down to single molecule conductors.^18−26^ This noise plays a central role in both fundamental research and technology since it contains useful information on device structure, material properties, and electron transport mechanisms. However, it may also obscure the signal in electronic devices and limit precision measurements. Electronic flicker noise originates from time-dependent resistance fluctuations that can be generated by various mechanisms, including charge trapping–detrapping and scattering of mobile charge carriers by defects and impurities with time-dependent scattering cross sections.^1,2,6,7,9−26^ Flicker noise is observed when a voltage difference or current bias is applied across a conductor, revealing the mentioned resistance fluctuations, though it can also be detected by temporal currents at thermal equilibrium.^27,28^

Here, we report an unknown version of flicker noise that is observed when a temperature difference is applied across a nanoscale conductor. This noise, termed here “delta-T flicker noise”, is demonstrated in atomic and molecular junctions subjected to temperature differences in the absence of a voltage bias or net current. The properties of the revealed delta-T flicker noise are examined in view of a theoretical model for quantum coherent transport while taking into account the impact of dynamic scatters at the metallic contacts. We find that delta-T flicker noise exhibits a quadratic dependence on the temperature difference, yet it is insensitive to the average temperature of the junction. Importantly, we verify that regular flicker noise, which is expected when a thermoelectric voltage is generated due to temperature differences,^29^ is negligible in the junction and cannot explain the probed noise.

Delta-T flicker noise can be an unwanted effect in electronic devices that suffer from unintentional temperature gradients. Such gradients are a growing concern for miniaturized modern electronics, where efficient heat dissipation becomes challenging.^30−33^ Furthermore, in superconducting qubit circuits, electronic flicker noise is a known performance-limiting factor.^6,34^ In view of our findings, finite temperature gradients in the vicinity of such qubits can lead to undesirable delta-T flicker noise. Thus, this overlooked noise contribution should be considered when designing and fabricating modern electronic devices. Measurements of temperature differences at the nanoscale are important for studying and regulating heat transport, heat dissipation, and energy conversion at the nanoscale. However, such measurements are technically challenging and typically require the design and fabrication of specialized temperature detectors.^35−37^ Delta-T flicker noise can serve as a simple probe for temperature differences in miniaturized systems down to atomic-scale conductors. The recently found frequency-independent delta-T noise (here, it will be called delta-T white noise)^38^ can also probe temperature differences at the nanoscale but *only* when the average temperature is known. The abundance of electronic flicker noise and its high magnitude at low frequencies can therefore open the door for a very general and accessible detection of temperature differences by the found delta-T flicker noise with no special setup requirements and in a large variety of miniature conductors and devices, regardless of their architecture, material, and dimensions.

Electronic flicker noise in nanoscale conductors is typically generated due to fluctuating scatters (e.g., defects, impurities, and adsorbates with time-dependent scattering cross-sections) near or inside the conductor, and it can be observed in the charge current when a voltage bias is applied. In the framework of Landauer formalism for quantum transport, considering dynamic scatters and assuming an energy-independent transmission probability, this flicker noise has the form of^25^
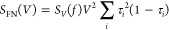
1defining *S*_*V*_(*f*) ≡ 2G_0_^2^ Φ(*f*), where Φ(*f*) is the power spectrum of reflection amplitudes due to the mentioned fluctuating scatters, *f* is the noise frequency, *G*_0_ ≅ 1/13 kΩ is the conductance quantum, *V* is the applied voltage, and *τ*_*i*_ is the transmission probability at the Fermi energy of the *i*th transmission channel. These channels are the transmission modes available for wave-like electrons crossing a quantum coherent conductor in some analogy to electromagnetic wave modes in a waveguide. As is clearly seen, at zero voltage, *S*_FN_(*V*) is nullified. Interestingly, another expression can be derived for flicker noise that is probed when a temperature difference is generated across a conductor (see Supporting Information, Section 1). Based on Landauer formalism for electron transport, yet with time dependent scattering probabilities, this noise adopts the form
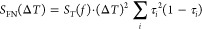
2with *S*_*T*_(*f*) ≡ 8(*G*_0_*k*_B_/*e*)^2^ Φ̃(*f*), where *k*_B_ is the Boltzmann’s factor, *e* is the electron charge, and Δ*T* is the temperature difference between the hot and cold sides of the conductor. The power spectrum function Φ̃(*f*) depends on energy derivatives of the reflection amplitude by dynamic scatters, evaluated at the Fermi energy. In contrast to a voltage bias that promotes a net current in one direction and allows flicker noise observation according to eq 1, a temperature difference promotes opposite and ideally equal currents between the electrodes with a *zero* net charge current. In this situation, the delta-T flicker noise (eq 2) can be observed in the absence of a voltage or a net current across a conductor. For an extended theoretical treatment of delta-T flicker noise that includes the diffusive regime, see Supporting Information, Section 1.

To experimentally track the mentioned delta-T flicker noise in quantum conductors, we apply a temperature difference across molecular junctions based on hydrogen molecules admitted between two opposite gold (Au) electrode tips.^25,38^ With the aid of a break-junction setup (Figure 1a),^39,40^ we can control the distance between the Au tips in subangstrom resolution, such that a variety of atomic-scale junctions decorated with hydrogen can be prepared in a base temperature of 4.2 K. The presence of hydrogen widens the conductance range of these junctions below the ∼0.75 *G*_0_ minimal conductance of bare single-atom Au junctions (Supporting Information, Figure S2),^39^ thus allowing us to characterize our measurements in view of eq 2, in a wider conductance range. According to the Landauer formalism for quantum transport, the conductance (*G*) is given by *G*_0_ times the sum of transmission probabilities for each channel, *G* = *G*_0_∑_*i*_τ_i_. In Au/hydrogen junctions, the conductance up to 1 *G*_0_ is given by one dominant channel along with very minor contributions from secondary channels,^39,41,42^ such that *G* ≅ *G*_0_τ_1_. Therefore, noise measurements for junctions with conductance below 1 *G*_0_ can be conveniently compared to the expected τ_*i*_^2^(1 – *τ*_*i*_) dependence of the delta-T flicker noise. To measure the noise in junctions subjected to a temperature difference, we heat one of the two electrodes and detect the temperature at each electrode by a thermometer located near the electrode tip. To find the temperature of each electrode in a nanoscale proximity to the junction, the thermometers are calibrated using the junction’s thermal (Johnson–Nyquist) noise^43−45^ at different temperatures, when no temperature difference is applied (see ref (38) and Supporting Information, Section 2).

**Figure 1:**
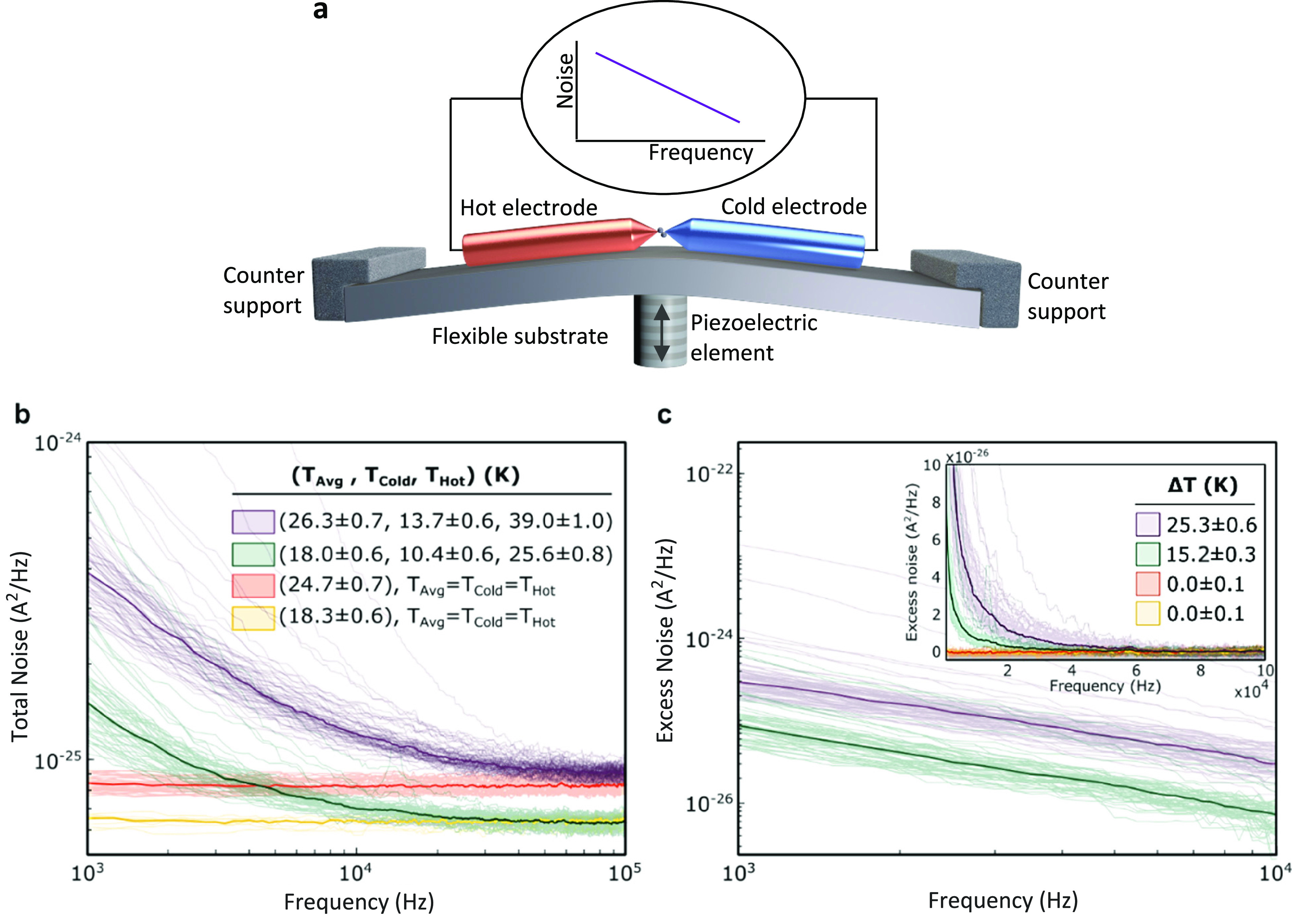
Experimental setup and measured noise at different temperature differences. (a) Illustration of the break junction setup and the measured flicker noise on a log–log scale. (b) Total noise as a function of the frequency measured in Au/hydrogen junctions. *T*_Hot_ and *T*_Cold_ are the different temperatures at opposite sides of the junctions, listed along with the average temperature *T*_Avg_. When no temperature difference is applied, only thermal (Johnson–Nyquist) noise is detected (yellow and orange curves). However, at a finite temperature difference an additional flicker noise contribution is found. (c) Excess noise (practically delta-T flicker noise) as a function of frequency, obtained after subtracting thermal noise and delta-T white noise^38^ from the total noise shown at (b). In the main panel of (c), excess noise is presented only for the two cases maintained at a finite temperature difference, while all four cases are presented on a linear scale in the Inset. The listed temperature differences Δ*T* correspond to the temperatures mentioned in (b). In each of the four temperature combinations, the noise data were collected for an ensemble of junctions with a zero-bias conductance in the range of 0.7–0.8 *G*_0_, with their median highlighted by thicker curves.

We start by looking for possible flicker noise that is probed when a temperature difference is applied across Au/hydrogen junctions. Figure 1b presents examples for the total noise measured in different junctions with conductance of 0.7–0.8 *G*_0_ at four different average temperatures, *T*_Avg_, and temperature differences, Δ*T*, (Figure 1c, inset) between the Au electrodes. Focusing first on the two cases with no applied temperature difference (orange and yellow curves), we find a median white noise that is equal to the expected thermal noise,^45^*S*_TN_ = 4*k*_B_*TG*, for these junctions when considering their conductance (0.77 *G*_0_ and 0.78 *G*_0_ for the orange and yellow curves, respectively) and temperatures (specified in Figure 1b). In contrast, when a temperature difference is applied across the junctions (green and purple curves), a new frequency-dependent noise contribution can be detected. This noise component is better seen in Figure 1c, once we subtract the frequency-independent noise contributions at the measured range (essentially, thermal noise and delta-T white noise^38^) from the total noise to get the frequency-dependent excess noise, or flicker noise. In the absence of a temperature gradient, no excess flicker noise is found and the signal is scattered around zero (Figure 1, inset), reflecting the uncertainty of the measurement. However, when a temperature difference is applied, a flicker noise with a 1/*f*^α^ ^12^ dependence on frequency is seen. This flicker noise is characterized by α ≅ 1 (Supporting Information, Figure S3), similar to the α of electronic flicker noise measured under a voltage bias across Au/hydrogen junctions.^25^ The revealed noise in Figure 1c cannot be detected in the absence of a temperature difference, and it is larger for a larger temperature difference. Therefore, we identify it as a flicker noise that is observed in the presence of a temperature difference across a nanoscale conductor, termed above as delta-T flicker noise.

To characterize the properties of delta-T flicker noise in view of eq 2, we integrate the noise in the range of 10^3^–10^4^ Hz, and we present it as a function of conductance in Figure 2a. Each data point is obtained for a different Au/hydrogen junction, experiencing a fixed temperature difference, Δ*T* = 15.2 ± 0.3 K with the average temperature, *T*_Avg_ = 18.0 ± 0.6 K. Junctions with conductance of several *G*_0_ are prepared by squeezing the two electrode tips (Figure 1a) against each other to obtain a junction cross-section of several Au atoms, contaminated with hydrogen. For a larger number of atoms in the junction, the conductance is larger, and in turn, the number of available transmission channels is higher. Note that whenever the channels are fully close (*τ*_*i*_ = 0) or fully open (*τ*_*i*_ = 1) their contribution to the noise is expected to be nullified according to eq 2. This is best seen by the reduction of the measured noise in Figure 2a close to 0 and 1 *G*_0_ for junctions that are dominated by a single channel. For a larger conductance, the effect is not pronounced due to the growing contribution of partially open channels.^25,39,41,42^ The distribution of the delta-T flicker noise as a function of conductance in Figure 2a is a consequence of its Σ_*i*_τ_*i*_^2^(1 – τ_*i*_) dependence on the number of channels *i* and their transmission probabilities *τ*_*i*_. This dependence is identical to that of the voltage flicker noise (eq 1) found when a voltage is applied across atomic and molecular junctions.^21,27^

**Figure 2:**
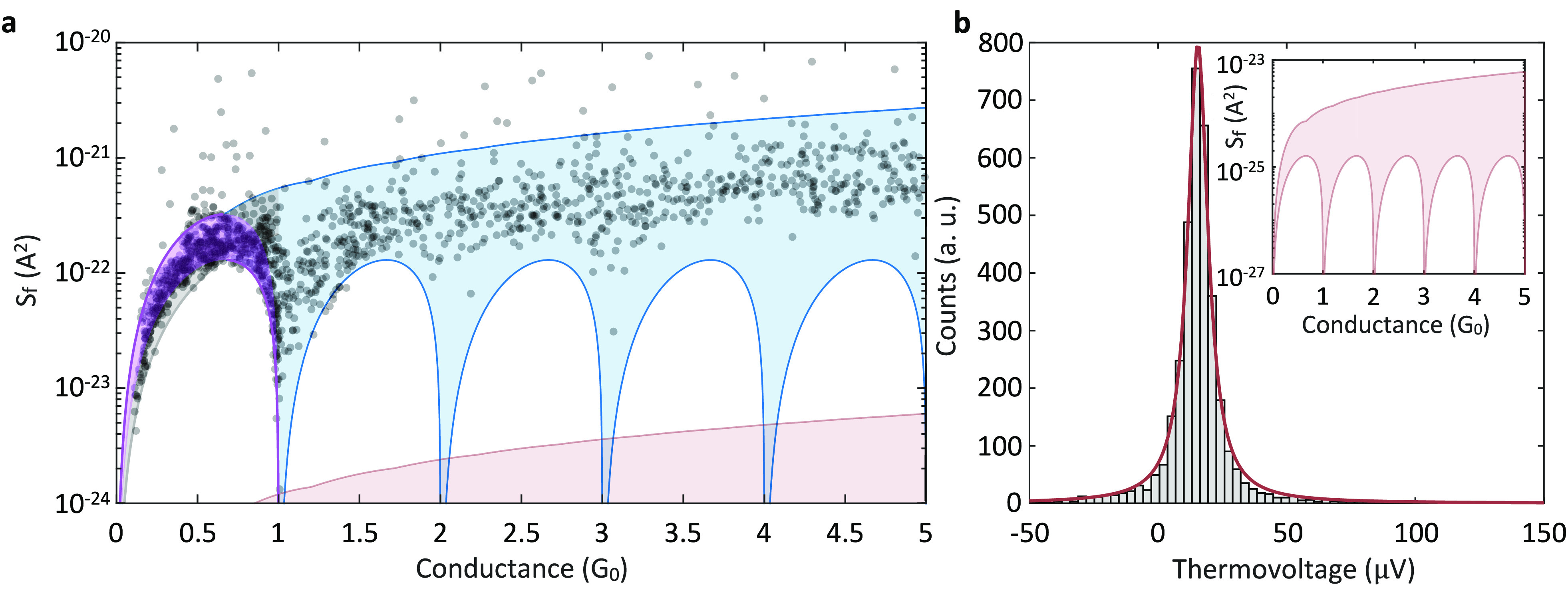
Flicker noise at a finite temperature difference and thermovoltage. (a) Excess noise integrated in 10^3^–10^4^ Hz, denoted as *S*_f_, as a function of conductance. Each data point (semitransparent black circle) is measured for a different Au/hydrogen junction realization at Δ*T* = 15.1 ± 0.3 K and *T*_Avg_ = 18.0 ± 0.6 K. The purple curves are fits of eq 2, considering a single channel below 1 *G*_0_, with τ_1_ = *G*/*G*_0_, yielding *S*_*T*_^max^ = 2.18 × 10^–21^ A^2^ and *S*_*T*_^min^ = 8.77 × 10^–22^ A^2^ (the scattered highest 5% of the points are ignored). Based on the found *S*_*T*_^max^, *S*_*T*_^min^, the purple region is the delta-T flicker noise range for junctions with a single transmission channel,^25^ the lower boundary of the blue region provides the lower limit for the noise expected for an ideal sequential opening of channels (see text), whereas its upper limit indicates the maximal noise expected for any number of channels. The gray region is the range of noise below 1 *G*_0_ that is expected for junctions with two equal transmission channels. The marked pink area is the estimated voltage flicker noise due to the measured thermovoltage presented in (b). (b) Histogram of the measured total thermoelectric voltage built in the junctions examined in (a), with a peak at 18 ± 2 μV (see Supporting Information, Section 2 and ref (47)). Inset: Calculated voltage flicker noise due to a thermovoltage of 18 μV, as also presented in (a). The calculation is based on maximal and minimal prefactors determined by measuring voltage flicker noise at different applied voltages in Au/hydrogen junctions (see Supporting Information, Figure S4).

In what follows, we focus on the main characteristics of the observed data distribution in Figure 2a. As mentioned, the delta-T flicker noise and the regular voltage flicker noise share identical characteristics when it comes to their dependence on transmission and therefore on conductance. An elaborated explanation on this dependence, including a theoretical derivation, can be found in ref (25). Here, to examine this dependence, we first find the prefactor *S*_*T*_(*f*)·(Δ*T*)^2^ relevant for Figure 2a (averaged for the range of 10^2^–10^3^ Hz). This prefactor provides the noise amplitude, and it is affected by the characteristics of the fluctuating scatters via Φ̃(*f*). In the experiments, the junctions are squeezed up to conductance of tens of *G*_0_ in different junction realizations to promote sampling of different junction geometries, including different distributions of fluctuating scatters near the junction’s constriction. This leads to a range of values for the prefactor between the extremums *S*_*T*_^min^ and *S*_*T*_^min^ that can be found by fitting eq 2 to the measured maximal and minimal data below 1 *G*_0_, assuming a single channel, with τ = *G*/*G*_0_ (purple curves). For the examined ensemble of junctions in Figure 2, *S*_*T*_^min^ = 2.18 × 10^–21^ A^2^ and *S*_*T*_^min^ = 8.77 × 10^–22^ A^2^. Using these prefactors and eq 2, we can now find the minimal bound for the expected noise (bottom blue curve) for an ideal sequential opening of channels. Namely, one channel is gradually opened between 0 and 1 *G*_0_ with τ_1_ = *G*/*G*_0_, then another channel is gradually opened between 1 and 2 *G*_0_ with τ_2_ = *G*/*G*_0_ – 1, while the first channel remains fully open with τ_1_ = 1, etc.), as expected for quantized conductance in an ideal point contact.^46^ Practically, for Au junctions (with or without hydrogen) the opening pattern slightly deviates from a strict sequential opening of channels since more than one channel is partially open at a given conductance above 1 *G*_0_.^25,39,41,42^ This leads to a higher minimal bound for the delta-T flicker noise in realistic junctions, as evident here by the measured lowest data points in Figure 2a for conductance larger than 1 *G*_0_. The top blue curve indicates the expected upper bound for the delta-T flicker noise, and data points above this bound indicate other noise contributions beyond the delta-T flicker noise (e.g., due to junction instability). Indeed, the vast majority of data points appear below this curve. Interestingly, along the theoretical upper bound the channels have the same contribution (τ_1_ = τ_2_ = ... = *τ*_*N*_ = *G*/(*NG*_0_)).^25^ The semitransparent blue region is therefore the expected distribution of delta-T flicker noise, which is confined by these two blue limits for any number of channels at a given conductance. A similar data analysis was carried out in the recent study of the quantum flicker noise^25^

Since the thermoelectric effect can generate a voltage when a temperature difference is applied,^48^ it is important to verify that the measured noise is not a regular voltage flicker noise found as a consequence of a built-up thermovoltage. Figure 2b presents a histogram of the thermovoltage measured across the ensemble of Au/hydrogen junctions for which the noise data in Figure 2a was measured, at a temperature difference of Δ*T* = 15.2 ± 0.3 K (see Supporting Information, Section 2). The peak indicates a most probable thermovoltage of 18 ± 2 μV, and the distribution is ascribed to variations in the fine structure of the different fabricated junctions.^49^ The expected voltage flicker noise due to the generated thermovoltage is found using eq 1 and presented in Figure 2a (pink) for the sake of comparison with the flicker noise data measured under temperature difference and in more detail in Figure 2b, inset. This noise is 2 orders of magnitude smaller than the flicker noise measured under the mentioned temperature difference and cannot explain its origin.

We now turn to examine the influence of the temperature on the studied flicker noise. Figure 3a shows the probed delta-T flicker noise as a function of conductance for various temperature differences (left horizontal axis) and average temperatures (listed in Figure 3b,c). To obtain the effect of temperature, Figure 3b presents the median of the noise of Figure 3a as a function of the average temperature in the conductance range of 3.5–4.4 *G*_0_. The figure shows a larger noise magnitude for a higher average temperature. However, the temperature difference is not identical at each average temperature. To remove the effect of this variable, Figure 2c presents normalized data obtained by dividing the data from Figure 3b by the relevant (Δ*T*)^2^ value for each average temperature. Interestingly, the normalized noise does not depend on the average temperature. We can thus conclude that the increase in amplitude in Figures 2a,b arises from the increase in the temperature difference, as stated by eq 2, and not by the rise in the average temperature. These findings are in a sharp contrast to the behavior of the previously reported delta-T white noise,^38^ a version of current noise that depends on both temperature difference and average temperature (∼(Δ*T*)^2^/*T*_Avg_). The absence of temperature dependence in the delta-T flicker noise arises due to the approximate cancellation of two effects within Φ̃(*f*) when increasing the average temperature: enhancement of the directional opposite charge currents and suppression of the time scale associated with scattering processes. For more details, see Supporting Information, Section 1.

**Figure 3:**
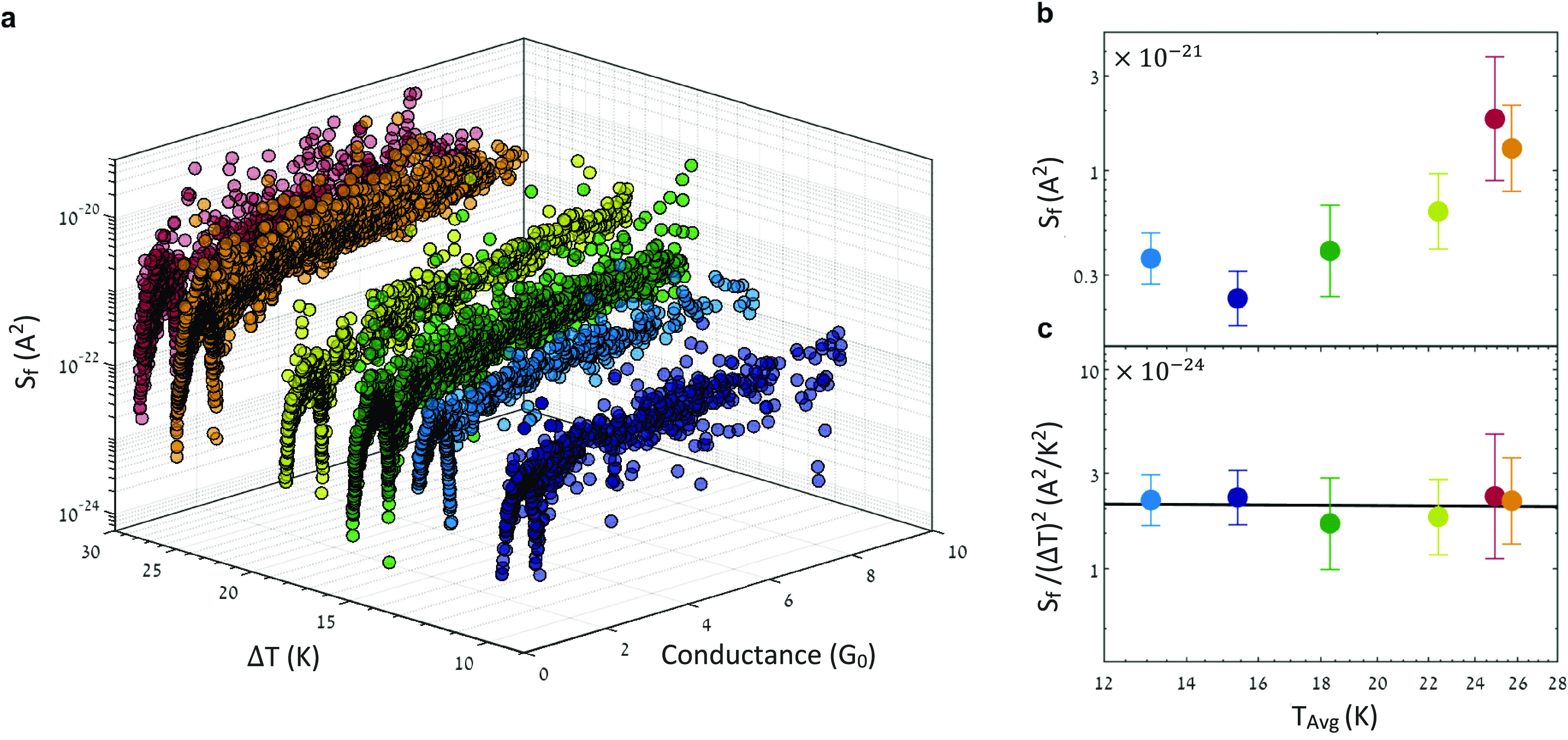
Flicker noise dependence on the temperature difference and average temperature. (a) Integrated excess noise as a function of conductance and temperature difference. Each data point is measured for a different Au/hydrogen junction realization. (b) Integrated excess noise as a function of average temperatures in the conductance range of 3.5–4.4 *G*_0_. The noise seems to be higher for a larger average temperature; however, the temperature difference is different at each average temperature. This extra variable is accounted in (c). (c) Integrated excess noise divided by the relevant (Δ*T*)^2^ as a function of average temperature. To examine the influence of the average temperature on the noise, the expected dependence on (Δ*T*)^2^ according to eq 2 is nullified by dividing the noise in (b) with this variable and presenting it in (c). No temperature dependence is observed in the considered range. The median noise is presented in (b) and (c) with its standard deviation.

The difference between delta-T white noise^38^ and delta-T flicker noise has important practical implications. Both noise contributions can be used to probe temperature differences. However, extracting temperature differences using delta-T white noise is possible only when the average temperature is known, while delta-T flicker noise has the advantage of probing temperature differences without the need to probe independently the average temperature. Considering the abundance of flicker noise and its experimental accessibility due to its high magnitude at low frequencies, we expect that delta-T flicker noise can be an attractive probe for temperature differences in nanoscale electronic conductors and devices. Such a probe is especially relevant in modern electronics, for which inefficient heat dissipation at the nanoscale may lead to unwanted temperature differences across nanoscale electronic components. Probing temperature differences across nanoscale conductors and devices is also central to the study of heat transport and energy conversion at the nanoscale. The detection of temperature differences is more challenging across nanoscale systems and usually requires sophisticated and expensive thermometry. However, this difficulty can be avoided using delta-T flicker noise.

Turning to fundamental aspects of noise, electronic flicker noise is resistive noise. A voltage bias or a temperature difference can either stimulate these time-dependent resistance fluctuations or merely probe resistance fluctuations that are already activated, for example, by thermal energy. Even in the absence of voltage or temperature gradients, thermal energy at a finite temperature can lead to time-dependent current fluctuations (thermal noise) that in turn can probe thermally activated resistance fluctuations (i.e., thermal equilibrium flicker noise^27,28^) or concurrently stimulate and probe it. We therefore distinguish between flicker noise forms that are activated and probed or merely probed by (i) voltage,^1^ (ii) temperature,^27^ and based on our findings, (iii) temperature differences. This classification has some analogy to the classification of white noise to shot noise,^50^ thermal noise,^43−45^ and delta-T white noise,^38^ which are activated and probed by the three mentioned stimuli (i–iii), respectively. However, the latter three noise versions are associated with time-dependent current fluctuations, in contrast to resistive flicker noises. In view of the above and as summarized in Table 1, we put forward the delta-T flicker noise as the missing noise form in a family of flicker noises, now including three members, those probed by voltage, temperature, and temperature difference.

**Table 1:** White Current Noise and Flicker Resistance Noise, Classified by Their Stimulus/Probe

Stimulus/Probe	White current noise	Flicker resistance noise
voltage	shot noise^50^	voltage flicker noise^1^
temperature	thermal noise^43−45^	thermal equilibrium flicker noise^27^
temperature difference	delta-T (white) noise^38^	delta-T flicker noise

## Supporting Information Available

Theory section containing theoretical derivation of the delta-T flicker noise, comparison to voltage flicker noise, flicker noise in the diffusive regime, and thermovoltage in the Landauer limit; experimental section containing sample preparation, conductance measurements, junction characterization, temperature measurements, flicker noise measurements at finite temperature differences, flicker noise measurements at a finite current bias, and thermovoltage measurements (PDF)
