# Supplementary material for: Delta-T Flicker Noise Demonstrated with Molecular Junctions
Source: Nano Lett. 2024 Jan 31;24(6):1981–7. doi: 10.1021/acs.nanolett.3c04445 (PMC10870783; doi:10.1021/acs.nanolett.3c04445)
Supplement: Supplementary file 1 — nl3c04445_si_001.pdf [file nl3c04445_si_001.pdf]

Supporting Information: Delta-T flicker noise demonstrated with molecular junctions

Ofir Shein-Lumbroso,[1] Matthew Gerry[$,2] Abhay Shastry[$,3] Ayelet Vilan,[1] Dvira Segal[*,2,3] and Oren Tal[*1]

[1]*Department of Chemical and Biological Physics, Weizmann Institute of Science, Rehovot 7610001, Israel*
[2]*Department of Physics, 60 Saint George St., University of Toronto, Toronto, Ontario, M5S 1A7, Canada*
[3]*Department of Chemistry and Centre for Quantum Information and Quantum Control, University of Toronto, 80 Saint George St., Toronto, Ontario, M5S 3H6, Canada*

(Dated: December 21, 2023)

[$]M.G. and A.S. contributed equally to this paper

[*] Corresponding authors
Dvira Segal, e-mail: dvira.segal@utoronto.ca
Oren Tal, e-mail: oren.tal@weizmann.ac.il

CONTENTS

SECTION 1: THEORY 2

I. Theory of the delta-T flicker noise 2
A. Transmission function with fluctuating defects 2
B. Power spectrum of the current fluctuations 3
C. Separable form of the return amplitudes 5
D. Time-dependent contact 6
E. Temperature dependence of the flicker noise 6

II. Comparison to voltage flicker noise 7

III. Flicker noise in the diffusive regime 7

IV. Thermovoltage in the Landauer limit 8

SECTION 2: EXPERIMENT 9

V. Sample preparation 9

VI. Conductance measurements 9

VII. Characterization of Au and Au/hydrogen junctions 10

VIII. Temperature measurements 10

IX. Measurements of flicker noise at finite temperature differences 10

X. Measurements of Flicker noise at a finite current bias 11

XI. Thermovoltage measurements 11

References 12

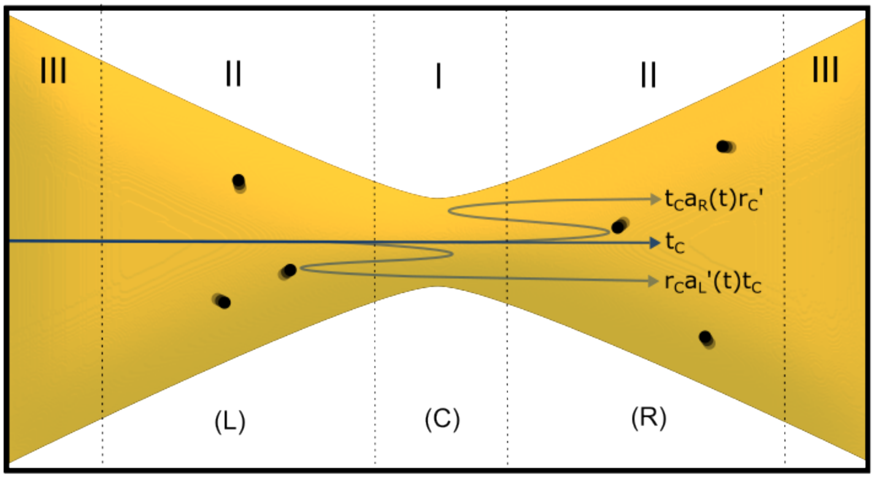

FIG. S1. Illustration of an atomic-scale junction through which charge current fluctuations were measured. The flicker noise is understood to occur as a result of coherent electrons scattering off of fluctuating defects, depicted as black dots, in the interface zones. The center contact region (C) is labelled (I), while the interface zones (L and R) are labelled (II) and the bulk metals (III). The arrows representing each possible path (no scattering by fluctuating defects at the interfaces, scattering by such defects either on the left or the right interfaces) are labelled with their respective amplitudes.

SECTION 1: THEORY

I. THEORY OF THE DELTA-T FLICKER NOISE

This section describes the modeling and derivation of the delta-T flicker noise. We begin in Sec. I A with the physical picture underlying the delta-T flicker noise in nanoscale junctions. The power spectrum of the noise is derived in Sec. I B, with Eqs. (S18)-(S19) as the working expressions. Additional simplifications to the power spectrum expression are detailed in Sec. I C, along with a discussion on the expected temperature dependence given in Sec. I E.

A. Transmission function with fluctuating defects

In the quantum-coherent limit, we use the Landauer-Büttiker formalism to describe the charge current through the contact,

$$I(t) = \frac{2e}{h}\int_{-\infty}^{\infty} d\epsilon\, \mathcal{T}(\epsilon, t)[f_L(\epsilon) - f_R(\epsilon)], \tag{S1}$$

where $f_v(\epsilon) = \{\exp[(\epsilon - \epsilon_F)/k_B T_v] + 1\}^{-1}$ denotes the Fermi-Dirac distribution for the v electrode with temperature T_v. The Fermi level, ϵ_F, is assumed common between the two electrodes.

We consider here charge transport through an atomic or molecular contact between two metal electrodes, with the possibility for transported electrons to scatter off of fluctuating defects in two "interface zones" to the left and right of the contact. The time dependence of the transmission, $\mathcal{T}(\epsilon, t)$, which arises due to these defects, is characterized by a timescale much slower than that of electrons tunnelling through the junction.

In particular, the transmission can be derived from scattering theory with transmission and reflection amplitude matrices for the central atomic-scale contact region (C), $\boldsymbol{t}_C$, $\boldsymbol{t}'_C$, $\boldsymbol{r}_C$, and $\boldsymbol{r}'_C$, as well as for each interface zone, $\boldsymbol{t}_v$, $\boldsymbol{t}'_v$, $\boldsymbol{a}_v$, and $\boldsymbol{a}'_v$. These matrices are of size $N \times N$, where N is the number of transmission channels that are considered. Note that the unprimed symbols correspond to the case where electrons come from the left and the primed symbols correspond to the case where they come from the right. Taking the return amplitudes due to scattering at the interface zones to be relatively small, we can treat the effect of scattering as a perturbation and include contributions only up to first order in the matrix elements $\boldsymbol{a}_{v,ii}$. As such, there are three contributions to the transmission function for each transmission channel: the dominant contribution corresponding to the case where no scattering occurs at the interfaces, as well as a contribution from the case where an electron reflects off the contact (amplitude $\boldsymbol{r}_{C,ii}$), scatters off of a defect in the left interface zone and then travels through the contact (amplitude $\boldsymbol{r}_{C,ii}\boldsymbol{a}'_{L,ii}\boldsymbol{t}_{C,ii}$), and one from the case where an electron initially travels through the contact, scatters off a defect on the right, and reflects again off the contact (amplitude $\boldsymbol{t}_{C,ii}\boldsymbol{a}_{R,ii}\boldsymbol{r}'_{C,ii}$). These three scenarios are depicted in Fig. S1. We get, explicitly

writing the energy and time dependence of these amplitudes as appropriate[1],

$$\begin{aligned}\mathcal{T}(\epsilon,t) &\simeq \sum_{i=1}^{N}\tau_i(\epsilon)\left[1+2\mathrm{Re}\left(\boldsymbol{r}_{C,ii}(\epsilon)\boldsymbol{a}'_{L,ii}(\epsilon,t)+\boldsymbol{a}_{R,ii}(\epsilon,t)\boldsymbol{r}'_{C,ii}(\epsilon)\right)\right] \\ &= \mathcal{T}_{const}(\epsilon)+\delta\mathcal{T}(\epsilon,t) \end{aligned} \tag{S2}$$

where each $\tau_i \equiv |t_{C,ii}|^2 = |t'_{C,ii}|^2$, representing the transmission probability for a given channel, i, consistent with the approximation that the transmission and reflection matrices are diagonal[1]. As mentioned above, this expression was constructed by assuming a small return amplitude at the interface zones. However, it can be generalized by revisiting the exact scattering formula[1], and including higher order scattering processes. We identify a time independent (constant) average value for the transmission probability as well as a fluctuating contribution due to the fluctuating scatterers in the interface zones,

$$\begin{aligned}\mathcal{T}_{const}(\epsilon) &= \sum_{i=1}^{N}\tau_i(\epsilon), \\ \delta\mathcal{T}(\epsilon,t) &= 2\sum_{i=1}^{N}\tau_i(\epsilon)\mathrm{Re}\left(\boldsymbol{r}_{C,ii}(\epsilon)\boldsymbol{a}'_{L,ii}(\epsilon,t)+\boldsymbol{a}_{R,ii}(\epsilon,t)\boldsymbol{r}'_{C,ii}(\epsilon)\right). \end{aligned} \tag{S3}$$

The time independent (constant) transmission is also denoted (Main text) by $\tau(\epsilon) \equiv \sum_{i=1}^{N}\tau_i(\epsilon)$. The expressions in (S3) may be inserted into Eq. (S1) to get $I(t) = \langle I\rangle + \delta I(t)$, with the fluctuating contribution to the current fluctuations given by,

$$\delta I(t) = \frac{2e}{h}\int_{-\infty}^{\infty} d\epsilon\, \delta\mathcal{T}(\epsilon,t)[f_L(\epsilon)-f_R(\epsilon)]. \tag{S4}$$

Inspecting Eq. (S3), we observe that these current fluctuations arise from two scenarios corresponding to the two terms inside the summation. In the first, incoming electrons reflect off the contact, reflect again off of a defect in the left interface zone to continue along in their original direction, and then pass through the contact. In the second, electrons pass through the contact initially, reflect off of a defect in the interface zone, and then reflect off the contact to continue travelling forward. In either case, we only consider situation in which exactly one scattering event takes place in the interface zones, reflecting the small magnitude of reflection amplitudes $\boldsymbol{a}_{v,ii}$ and $\boldsymbol{a}'_{v,ii}$ and their subsequent treatment as perturbations[1,2].

We note that in Ref.[3], it was shown that the voltage fluctuations are related to conductance fluctuations, which are ultimately caused by the random fluctuations in the charge traps. In particular, it was demonstrated that $\Delta V/V = \Delta G/G$ for both Lorentzian (caused by a single trap) as well as $1/f$ (caused by multiple traps) line shapes. Similarly, our theoretical model predicts that current fluctuations result from conductance fluctuations. However, in our model, the conductance fluctuations arise from the fluctuations in the return amplitude, rather than fluctuations of the potential energy ϵ_0 of the quantum point contact in Ref.[3].

B. Power spectrum of the current fluctuations

Flicker noise describes the $1/f$ dependence of the power spectrum of the fluctuating current at low frequency f. To investigate the power spectrum, we consider a characteristic sample $\delta I(t)$ recorded in a time interval (0, t_m), and focus below on deriving the dependence of the power spectrum on the transmission channel distribution. The power spectrum is given by

$$S_{FN}(\Delta T) = \lim_{t_m\to\infty}\frac{1}{t_m}\left|\int_0^{t_m} dt\, \delta I(t)\exp(2\pi i f t)\right|^2, \tag{S5}$$

where the limit of long measurement time is taken. For a stationary process $\delta I(t)$, Wiener-Khinchin theorem relates the above expression to the current correlation function $\langle\delta I(t)\delta I(t+t')\rangle$[4]. We use $\delta I(t)$ as defined in Eq. (S4), and expand out $\delta\mathcal{T}(\epsilon,t)$,

$$\delta I(t) \simeq \frac{4e}{h}\sum_i \tau_i\int_{-\infty}^{\infty} d\epsilon\, \mathrm{Re}\left(\boldsymbol{r}_{C,ii}\boldsymbol{a}'_{L,ii}(\epsilon,t)+\boldsymbol{a}_{R,ii}(\epsilon,t)\boldsymbol{r}'_{C,ii}\right)(f_L(\epsilon)-f_R(\epsilon)). \tag{S6}$$

Note that the transmission and reflection amplitudes for the contact region do not vary significantly over the energy range around the Fermi energy ϵ_F, for which the difference $f_L - f_R$ takes on non-negligible values. As such, they are replaced by their values at ϵ_F and taken to be constant. We note that the only time dependence appears in the return amplitudes due to the fluctuating defects. Thus, we define,

$$\boldsymbol{a}_{v,ii}(\epsilon,f;t_m) \equiv \int_0^{t_m} dt\, \boldsymbol{a}_{v,ii}(\epsilon,t)\exp(2\pi i f t), \tag{S7}$$

and write the full expression for the power spectrum,

$$\begin{aligned} &S_{FN}(\Delta T) \\ &= 4\frac{G_0^2}{e^2}\lim_{t_m\to\infty}\frac{1}{t_m}\left|\sum_i \tau_i \mathrm{Re}\left[\int_{-\infty}^{\infty} d\epsilon\ \left(\boldsymbol{r}_{C,ii}\boldsymbol{a}'_{L,ii}(\epsilon,f;t_m)+\boldsymbol{a}_{R,ii}(\epsilon,f;t_m)\boldsymbol{r}'_{C,ii}\right)(f_L(\epsilon)-f_R(\epsilon))\right]\right|^2 \\ &= 4\frac{G_0^2}{e^2}\lim_{t_m\to\infty}\frac{1}{t_m}\left|\sum_i \tau_i \mathrm{Re}\left[\boldsymbol{r}_{C,ii}\int_{-\infty}^{\infty} d\epsilon\ \boldsymbol{a}'_{L,ii}(\epsilon,f;t_m)(f_L(\epsilon)-f_R(\epsilon))+\boldsymbol{r}'_{C,ii}\int_{-\infty}^{\infty} d\epsilon\ \boldsymbol{a}_{R,ii}(\epsilon,f;t_m)(f_L(\epsilon)-f_R(\epsilon))\right]\right|^2, \end{aligned} \tag{S8}$$

where $G_0 = 2e^2/h$ is the conductance quantum. To simplify the next steps, we define the quantity

$$A_{v,ii}(f;t_m) \equiv \int_{-\infty}^{\infty} d\epsilon\ \boldsymbol{a}_{v,ii}(\epsilon,f;t_m)(f_L(\epsilon)-f_R(\epsilon)). \tag{S9}$$

We will discuss how this integral may be evaluated later on. Note that this quantity captures the dependence of the power spectrum on the temperature difference due to the presence of the Fermi-Dirac distributions in the integral. We rewrite the expression for the power spectrum,

$$\begin{aligned} S_{FN}(\Delta T) &= 4\frac{G_0^2}{e^2}\lim_{t_m\to\infty}\frac{1}{t_m}\left|\sum_i \tau_i \mathrm{Re}\left[\boldsymbol{r}_{C,ii}A'_{L,ii}(f;t_m)+\boldsymbol{r}'_{C,ii}A_{R,ii}(f;t_m)\right]\right|^2 \\ &= 4\frac{G_0^2}{e^2}\sum_i \tau_i^2 \lim_{t_m\to\infty}\frac{1}{t_m}\left(\mathrm{Re}\left[\boldsymbol{r}_{C,ii}A'_{L,ii}(f;t_m)+\boldsymbol{r}'_{C,ii}A_{R,ii}(f;t_m)\right]\right)^2 \\ &= \frac{G_0^2}{e^2}\sum_i \tau_i^2 \lim_{t_m\to\infty}\frac{1}{t_m}\left(\boldsymbol{r}_{C,ii}A'_{L,ii}(f;t_m)+\boldsymbol{r}'_{C,ii}A_{R,ii}(f;t_m)+\mathrm{c.c.}\right)^2 \\ &\approx \frac{G_0^2}{e^2}\sum_i \tau_i^2 \lim_{t_m\to\infty}\frac{1}{t_m}2(1-\tau_i)\sum_{v=L,R}|A_{v,ii}(f;t_m)|^2 \\ &= 2\frac{G_0^2}{e^2}\sum_i \tau_i^2(1-\tau_i)\lim_{t_m\to\infty}\frac{1}{t_m}\sum_{v=L,R}|A_{v,ii}(f;t_m)|^2. \end{aligned} \tag{S10}$$

We arrived at the second line by neglecting correlations between different channels, which is consistent with the derivation of the transmission function. We arrive at the fourth line by keeping only contributions that remain nonzero in the limit of long measurement time. This includes neglecting correlations between the left and right interface zones.

The remaining task is to evaluate integrals of the form of Eq. (S9). In the case of a temperate difference $\Delta T = T_L - T_R$ and no voltage bias, the difference $f_L(\epsilon) - f_R(\epsilon)$ is a strictly odd function. Thus, only the odd parts of $\boldsymbol{a}_{v,ii}(\epsilon, f; t_m)$ contribute to the integral. We use an exact series expansion to evaluate integrals of this form,

$$\int_{-\infty}^{\infty} d\epsilon\ F(\epsilon)(f_2(\epsilon)-f_1(\epsilon)) = \int_{\mu_1}^{\mu_2} d\epsilon\ F(\epsilon) + 2\sum_{k\ \mathrm{odd}}\Theta(k+1)\left[(k_BT_2)^{k+1}F^{(k)}(\mu_2)-(k_BT_1)^{k+1}F^{(k)}(\mu_1)\right], \tag{S11}$$

where the summation is over positive odd integers $k = 1, 3, 5, ...$, $F^{(k)}(\mu)$ represents the kth order derivative of a function $F(\epsilon)$ evaluated at $\epsilon = \mu$, and the factor $\Theta(k + 1)$ is related to the Riemann Zeta function as,

$$\Theta(k+1) = \left(1-\frac{1}{2^k}\right)\zeta(k+1). \tag{S12}$$

Particular values of the Riemann Zeta function are $\zeta(2) = \frac{\pi^2}{6}$, $\zeta(4) = \frac{\pi^4}{90}$ and $\zeta(6) = \frac{\pi^6}{945}$. The expression (S9) is therefore given by,

$$A_{v,ii}(f;t_m) = \int_{-\infty}^{\infty} d\epsilon\ \boldsymbol{a}_{v,ii}(\epsilon,f;t_m)(f_L(\epsilon)-f_R(\epsilon)) = 2\sum_{k\ \mathrm{odd}}\Theta(k+1)\boldsymbol{a}^{(k)}_{v,ii}(\epsilon_F,f;t_m)\left[(k_BT_L)^{k+1}-(k_BT_R)^{k+1}\right]. \tag{S13}$$

We can rewrite Eq. (S13) in terms of the average temperature between the metals, $T_{Avg} = (T_L + T_R)/2$ and the temperature difference $\Delta T = T_L - T_R$, using,

$$(k_BT_L)^{k+1}-(k_BT_R)^{k+1} = (k_BT_{Avg})^{k+1}\left((k+1)\frac{\Delta T}{T_{Avg}}+\frac{1}{24}k(k^2-1)\left(\frac{\Delta T}{T_{Avg}}\right)^3+\mathcal{O}\left(\frac{\Delta T}{T_{Avg}}\right)^5\right). \tag{S14}$$

To leading order in ΔT, we get,

$$A_{v,ii}(f;t_m) = 2\sum_{k \text{ odd}} (k+1)\Theta(k+1)[(k_B T_{Avg})^k \boldsymbol{a}^{(k)}_{v,ii}(\epsilon_F, f; t_m)](k_B \Delta T). \tag{S15}$$

We define a quantity that captures the f-dependence of the contribution from each interface zone once the limit of long measurement time is taken,

$$\tilde{\Phi}_{v,ii}(f) = \lim_{t_m \to \infty} \frac{1}{t_m} \left| \sum_{k \text{ odd}} (k+1)\Theta(k+1)[(k_B T_{Avg})^k \boldsymbol{a}^{(k)}_{v,ii}(\epsilon_F, f; t_m)] \right|^2. \tag{S16}$$

We may finally return to the full expression for the power spectrum, to leading order in ΔT,

$$S_{FN}(\Delta T) = 8\frac{G_0^2}{e^2}(k_B \Delta T)^2 \sum_i \tau_i^2(1-\tau_i) \sum_{v=L,R} \tilde{\Phi}_{v,ii}(f) + \mathcal{O}(\Delta T^4). \tag{S17}$$

The validity of this first order approximation is discussed in Sec. I C. If we do not distinguish between the defect configurations of different channels, we may drop the index ii from the spectrum $\tilde{\Phi}_{v,ii}(f)$, giving,

$$S_{FN}(\Delta T) = S_T(f)(\Delta T)^2 \sum_i \tau_i^2(1-\tau_i), \tag{S18}$$

which is of the form of Eq. (2) in the main text describing the delta-T flicker noise, with the frequency dependence captured by

$$S_T(f) = 8\frac{G_0^2 k_B^2}{e^2} \sum_{v=L,R} \tilde{\Phi}_v(f). \tag{S19}$$

The low frequency behavior of $S_{FN}(\Delta T)$ is found to be determined completely by that of the $\tilde{\Phi}_v(f)$'s in the regime where leading-order contributions in ΔT suffice. This limit is discussed in Sec. I E. We observe a unique dependence of the flicker noise spectrum on the microscopic picture of transmission channels. As for the frequency (f) dependence, in the main text the flicker noise was experimentally collected in the frequency range $10^3 - 10^4$ Hz (Fig. 1). As shown in Ref.[1], flicker noise at this range can arise from a set of random telegraph noise sources with dwell times spanning 1 to 0.01 milliseconds.

C. Separable form of the return amplitudes

Further simplifications can be made in the special case where the return amplitude due to scattering processes at the interfaces takes on a separable form with respect to its dependence on time and on the energy of incoming electrons, i.e., $\boldsymbol{a}_{v,ii}(\epsilon, t) = a_{v,ii}(\epsilon)a_{ii}(t)$ (note also the time-dependence is of the same kind for $v = L$ and R). A lower case 'a' written with only a single argument is taken to depend only on that argument. In this case, Eq. (S9) simplifies to

$$A_{v,ii}(f, t_m) \to a_{ii}(f, t_m) \int_{-\infty}^{\infty} d\epsilon\, a_{v,ii}(\epsilon)(f_L(\epsilon) - f_R(\epsilon)), \tag{S20}$$

and the effect of the return amplitude's time-dependence may be treated separately from that of its dependence on the energy of incoming electrons. Through the Wiener-Khinchin theorem, the power spectrum $S_{FN}(\Delta T)$ can be obtained as the Fourier transform of the charge current autocorrelation function, $\langle \delta I(t)\delta I(t+t')\rangle$ (where the angle brackets denote the average over time t and the Fourier transform is taken with respect to t'). In this special case, this function's time dependence is captured entirely by the corresponding autocorrelation of the return amplitude's time dependence. We have that

$$\tilde{\Phi}_{v,ii}(f) = \Lambda_{v,ii}^2 \int_{-\infty}^{\infty} dt'\, \langle a_{ii}(t)a_{ii}(t+t')\rangle \exp(2\pi i f t'), \tag{S21}$$

where $\Lambda_{v,ii}$ is a factor that captures the impact of the dependence of $a_{v,ii}(\epsilon)$ on the energy of incoming electrons (i.e. via an equation similar to Eq. (S15) but with the time-dependence of the return amplitudes separated out and excluded).

With an understanding of the form of $a_{ii}(t)$, this model can then be used to obtain the observed $1/f$-behaviour of the power spectrum, for instance, if the return amplitudes vary in time as random telegraph signals[1,5].

Furthermore, if the forms of the energy dependent parts $a_{v,ii}(\epsilon)$ are known, the integral over energy values can be evaluated via the expansion,

$$\int_{-\infty}^{\infty} d\epsilon\, a_{v,ii}(\epsilon)(f_L(\epsilon) - f_R(\epsilon)) = 2\sum_{k \text{ odd}} \Theta(k+1) a^{(k)}_{v,ii}(\epsilon_F)\big[(k_B T_L)^{k+1} - (k_B T_R)^{k+1}\big]. \tag{S22}$$

We assume that scatters behave similarly at both interfaces, and for different channels, $a_{v,ii}(\epsilon) = a(\epsilon)$, and consider the model of Ref.[2], wherein the energy dependence amounts to a phase

$$a(\epsilon) = \exp(i(\epsilon - \epsilon_F)\mathcal{T}_{cl}/\hbar). \tag{S23}$$

This form reflects the coherent nature of electron transport between scattering events, with a characteristic timescale $\mathcal{T}_{cl}$ for electrons to scatter off defects in the interface zones and return to the contact. This timescale is determined through classical arguments and taken to be much faster than the timescale for the dynamics of the fluctuating defects themselves[2]. In this case, we have $a^{(k)}(\epsilon_F) = (i\mathcal{T}_{cl}/\hbar)^k$. Combining Eq. (S22) with Eq. (S14), we note that each term contains a factor $(ik_BT_{Avg}\mathcal{T}_{cl}/\hbar)^k$. Thus, the series converges in the case that $k_BT_{Avg}\mathcal{T}_{cl}/\hbar < 1$. For average temperatures on the order of $5-20\ K$, this corresponds to characteristic scattering times in the range of 0.2 picosecond or less.

The condition $k_BT_{Avg}\mathcal{T}_{cl}/\hbar < 1$ also permits the truncation of the series in Eq. (S22). If only the first term is kept, this amounts to taking the leading order approximation in ΔT, since the coefficients of the higher order terms in Eq. (S14) vanish for $k = 1$. The expression for the delta-T flicker noise power spectrum given in Eq. (S17) is valid in this regime.

D. Time-dependent contact

We briefly consider the case where the transmission and reflection coefficients associated with the contact region (C) itself exhibit time dependence. Accordingly, each $\tau_i \to \tau_i(t)$, and each $\boldsymbol{r}_{C,ii} \to \boldsymbol{r}_{C,ii}(t)$. The power spectrum at low frequency can be derived following a similar approach to that of Sec. I B. However, rather than just the Fourier transforms of the return amplitudes as defined in Eq. (S7), we must consider the Fourier transforms of the products $\tau_i(t)\boldsymbol{r}_{C,ii}(t)\boldsymbol{a}'_{L,ii}(\epsilon,t)$ and $\tau_i(t)\boldsymbol{a}_{R,ii}(\epsilon,t)\boldsymbol{r}'_{C,ii}(t)$, which we define as $\phi_{L,ii}(\epsilon,f;t_m)$ and $\phi_{R,ii}(\epsilon,f;t_m)$, respectively.

In analogy to Eq. (S9), we may define a quantity to capture the integration over energy values,

$$\tilde{A}_{v,ii}(f;t_m) = \int_{-\infty}^{\infty} d\epsilon\ \phi_{v,ii}(\epsilon,f;t_m)(f_L(\epsilon) - f_R(\epsilon)). \tag{S24}$$

Then, the power spectrum takes the form,

$$S_{FN}(\Delta T) = 2\frac{G_0^2}{e^2}\lim_{t_m\to\infty}\frac{1}{t_m}\sum_i\sum_{v=L,R}|\tilde{A}_{v,ii}(f;t_m)|^2. \tag{S25}$$

This summation over i is missing the factor $\tau_i^2(1-\tau_i)$ that we see when the transmission and reflection coefficients through the contact are constant. Instead, the effect of the transmission channel distribution on the noise spectrum has a complicated dependence on the behavior of the transmission and reflection amplitudes. This effect is captured in the quantities $\tilde{A}_{v,ii}(f;t_m)$, and its specific features cannot be known without knowledge of how the contact transmission and reflection amplitudes vary in time.

In analogy with Eq. (S15), one gets that

$$\tilde{A}_{v,ii}(f) = 2\sum_{k\ \mathrm{odd}}(k+1)\Theta(k+1)[(k_BT_{Avg})^k\boldsymbol{\phi}^{(k)}_{v,ii}(\epsilon_F,f)](k_B\Delta T). \tag{S26}$$

Assuming transmission and reflection amplitudes at the centre are about constant in energy we write (simplifying the notation by omitting the long integration time t_m),

$$\boldsymbol{\phi}^{(k)}_{v,ii}(\epsilon_F,f) \propto \int_{-\infty}^{\infty} df_1\tau_i(\epsilon_F,f_1)\boldsymbol{r}_{C,ii}(\epsilon_F,f_1)\boldsymbol{a}^{(k)}_{v,ii}(\epsilon_F,f-f_1). \tag{S27}$$

In this limit, scattering amplitudes at both the contact center and the scatterers at the the interface zones fluctuate, thus the power spectrum depends on their respective frequency transforms via a frequency *convolution*. However, if the dynamics of the contact is significantly slower than the dynamics of fluctuating defects, we can assume that $\tau_i(\epsilon_F,f)\boldsymbol{r}_{C,ii}(\epsilon_F,f) \propto \delta(f)$. This limit, once employed in Eqs. (S25)-(S27) reduces the power spectrum back to Eq. (S17).

E. Temperature dependence of the flicker noise

While we have identified in the delta-T flicker noise a strictly nonequilibrium phenomenon, with $S_{FN}(\Delta T)$ vanishing in the limit that the two metals are at equal temperature, it is interesting to consider the impact of the average temperature between the two metals T_{Avg}. For one, Eq. (S15) amounts to an expansion in powers of (k_BT_{Avg}). As such, one may naïvely expect a strong T_{Avg}-dependence of the power spectrum, possibly with very large contributions from higher-order terms in the summation.

It is worth noting, however, that the return amplitudes themselves may exhibit nontrivial temperature dependence that alter this behavior. For instance, we revisit the model in which the energy dependence is given by a phase, Eq. (S23).

In this case, k^{th}-order differentiation of the return amplitude with respect to energy (ϵ) brings down a factor of $(\mathcal{T}_{cl}/\hbar)^k$, and Eq. (S15) can be written as an expansion in powers of $(k_B T_{Avg}\mathcal{T}_{cl}/\hbar)$. The dependence of $\mathcal{T}_{cl}$ on temperature thus significantly impacts the overall temperature dependence of the power spectrum. For instance, one may argue that $\mathcal{T}_{cl}$ drops off approximately as $1/T_{Avg}$ on the basis that higher temperature increases the number of active scatterers in the interface zone, shortening the timescale for transported electrons to reach a scatterer. Such a dependence would completely eliminate the dependence of the power spectrum on T_{Avg} to leading order in ΔT.

We stress that this analysis does *not* ascribe different behavior to $a_{L,ii}(\epsilon)$ and $a_{R,ii}(\epsilon)$ due to the differing temperatures of the two metals, $T_L \neq T_R$. While these arguments may lead to the inference that the hotter metal has, on average, more active scatterers than the colder metal, we recall that we only consider contributions to the power spectrum to first order in the return amplitudes. Thus, as is apparent in Eq. (S6), our theory only captures effects associated with the total number of scattering events in either metal (we sum over contributions from the two sides); it is indifferent to whether the scattering occurs on the left or right. As such, the contribution to the power spectrum capturing the ΔT dependence may be written without reference to the indices L and R, as $\tilde{\Phi}_{ii}(f) = \sum_{v=L,R}\tilde{\Phi}_{v,ii}(f)$. The underlying cause of the delta-T flicker noise is reflected not in any difference between the behavior of defects in the two metals, but in the difference between the Fermi-Dirac distributions describing the incoming electrons from the two sides, bringing about effects associated with how the return amplitudes $\boldsymbol{a}_{v,ii}(\epsilon, t)$ vary in energy.

II. COMPARISON TO VOLTAGE FLICKER NOISE

The derivation of the delta-T flicker noise closely follows the approach taken in Ref.[1] to derive the power spectrum at low frequency for voltage flicker noise, in the absence of a temperature difference. However, in that case, the even (rather than odd) symmetry of the function $f_L(\epsilon) - f_R(\epsilon)$ makes the evaluation of the integral captured by $A_{v,ii}(f;t_m)$ much simpler. To leading order in the voltage bias V, $A_{v,ii}(f;t_m)$ is directly proportional to the value of $\boldsymbol{a}_{v,ii}(\epsilon, f; t_m)$ evaluated at the Fermi level $\epsilon = \epsilon_F$, see Eq. (S9). As such, the contribution to the power spectrum capturing the frequency-dependence can be written simply in terms of the Fourier-transformed return amplitudes, i.e.,

$$\Phi_{v,ii}(f) = \lim_{t\to\infty} \frac{1}{t_m} \left|\boldsymbol{a}_{v,ii}(\epsilon_F, f; t_m)\right|^2 . \tag{S28}$$

An interesting distinction between the voltage and delta-T flicker noise is that the latter describes a situation in which there is zero overall net charge current. While the flicker noise is a strictly nonequilibrium phenomenon, the flow of higher-energy electrons from the hot to cold metal is cancelled out by the flow of lower-energy electrons from cold to hot. This indicates that the energy dependence of the return amplitudes $\boldsymbol{a}_{v,ii}(\epsilon, t)$ plays a central role in giving rise to the flicker noise that is detected in this scenario. This is in contrast with the voltage case, where simply the fact that the return amplitude takes on a nonzero value at $\epsilon = \epsilon_F$ is sufficient to derive the phenomenon of voltage flicker noise.

Interestingly, this means that the delta-T flicker noise could be useful in transmission channel analysis even in situations in which the conductance $G = \sum_i \tau_i$ cannot be measured due to the overall average current summing to zero.

III. FLICKER NOISE IN THE DIFFUSIVE REGIME

The diffusive regime corresponds to a particular situation regarding the relative spatial scales of the contact and the mean free path of transported electrons. Namely, the length, L, of the contact region satisfies $l \ll L \ll Nl$, where l is the mean free path and N is the number of transmission channels. Results from random matrix theory show that Nl sets the spatial scale for electron localization. This regime is diffusive in the sense that the contact is much larger than the mean free path, however, collisions in this region are taken to occur without the loss of phase coherence[6].

Investigations of charge transport in the diffusive limit have uncovered a suppression of the *shot* noise to 1/3 of the Poisson value of $2e|V|G$[7]. This limit is characterized by a bimodal distribution over the transmission probabilities associated with the N channels, with many permitting only a small contribution to the conductance, $\tau_i \ll 1$, and some being nearly fully open $1 - \tau_i \ll 1$[6]. We consider the corresponding behavior of the flicker noise in this limit by evaluating the factor that captures its dependence on the transmission channel distribution,

$$S_{FN}(\Delta T) \propto \sum_i \tau_i^2(1-\tau_i) \approx \frac{G}{G_0}\left(\frac{\langle\tau^2\rangle}{\langle\tau\rangle} - \frac{\langle\tau^3\rangle}{\langle\tau\rangle}\right), \tag{S29}$$

where $G = G_0 \sum_i \tau_i \approx G_0 N\langle\tau\rangle$ is the conductance, with N the number of channels. Angle brackets denote an average over transmission channels i, with associated transmission probabilities τ_i given in terms of a randomly sampled channel-dependent

localization length greater than the mean free path but less than the size of the junction[7]. Results from random matrix theory give the relation

$$\frac{\langle \tau^p \rangle}{\langle \tau \rangle} = \frac{\Gamma(1/2)\Gamma(p)}{2\Gamma(p+1/2)}, \tag{S30}$$

which can be used to evaluate Eq. (S29),

$$\sum_i \tau_i^2 (1-\tau_i) \approx \frac{G}{G_0}\left(\frac{2}{3} - \frac{8}{15}\right) = \frac{2}{15}\frac{G}{G_0}. \tag{S31}$$

As such, in this limit, the power spectrum for flicker noise is directly proportional to the conductance G. In the case of delta-T flicker noise as derived in Eq. (S18) we find that

$$S_{FN}(\Delta T) \approx \frac{16}{15}\frac{GG_0}{e^2}(k_B \Delta T)^2 \sum_{v=L,R} \tilde{\Phi}_v(f). \tag{S32}$$

IV. THERMOVOLTAGE IN THE LANDAUER LIMIT

The thermoelectric effect can generate voltage in the presence of a temperature difference. This thermoelectric voltage can lead to the voltage flicker noise—as an additional flicker noise at a finite temperature difference. However, as showed in the main text, in the examined junctions the voltage flicker noise due to the thermoelectric voltage is markedly lower than the delta-T flicker noise. We summarize in this Section established results for coherent transport: (i) In the case of constant transmission function, the average charge current under a temperature difference is zero. (ii) The thermopower depends on the energy derivative of the transmission function, evaluated at the Fermi energy.

In the Landauer theory, the time-averaged charge current under a *temperature difference* ΔT, rather than a voltage-bias is given by Eq. (S1),

$$\langle I \rangle = \frac{2e}{h}\int_{-\infty}^{\infty} d\epsilon \langle \mathcal{T}(\epsilon)\rangle \left[\frac{1}{e^{\beta_L(\epsilon-\epsilon_F)}+1} - \frac{1}{e^{\beta_R(\epsilon-\epsilon_F)}+1}\right], \tag{S33}$$

with $\beta_v = 1/(k_B T_v)$. The time-averaged transmission function is given by $\langle \mathcal{T}(\epsilon)\rangle$, but henceforth, for simplifying notation we do not display the time-averaging brackets. Assuming an energy independent transmission function, which is a reasonable situation for gold junctions under low bias and small temperature differences, and shifting the energy integration we get

$$\langle I \rangle = \frac{2e}{h}\mathcal{T}(\epsilon_F)\int_{-\infty}^{\infty} d\epsilon \left[\frac{e^{\beta_R \epsilon} - e^{\beta_L \epsilon}}{(e^{\beta_L \epsilon}+1)(e^{\beta_L \epsilon}+1)}\right], \tag{S34}$$

which is zero given the odd symmetry of the integrand. The first nontrivial correction to this expression develops once allowing the transmission function to vary with energy, by building the Taylor expansion $\mathcal{T}(\epsilon) \approx \mathcal{T}(\epsilon_F) + \frac{\partial \mathcal{T}}{\partial \epsilon}\Big|_{\epsilon_F}\epsilon$. The charge current under temperature bias can now be evaluated as

$$\begin{aligned}\langle I \rangle &= \frac{2e}{h}\frac{\partial \mathcal{T}}{\partial \epsilon}\Bigg|_{\epsilon_F}\int_{-\infty}^{\infty} d\epsilon\epsilon \left[\frac{1}{e^{\beta_L \epsilon}+1} - \frac{1}{e^{\beta_R \epsilon}+1}\right] \\ &= \frac{2e}{h}\frac{\partial \mathcal{T}}{\partial \epsilon}\Bigg|_{\epsilon_F}\frac{\pi^2 k_B^2 T_{Avg}}{3}\Delta T \\ &= G_0 \mathcal{T}(\epsilon_F) S_{TP}\Delta T, \end{aligned} \tag{S35}$$

where $S_{TP} = \frac{1}{\mathcal{T}(\epsilon_F)}\frac{\partial \mathcal{T}}{\partial \epsilon}\Big|_{\epsilon_F}\left(\frac{\pi^2 k_B^2 T_{Avg}}{3e}\right)$ is the thermopower. The thermovoltage, which is the voltage countering the temperature bias to reach zero charge current is given by $V_{TP} \equiv S_{TP}\Delta T$. In the measured junctions (main text) using ΔT=15.1±0.3 K and T_{Avg}=18.0±0.6 K, the most probable thermovoltage was at $V_{TP} \approx 18 \pm 2\ \mu V$, and the resulting thermovoltage flicker noise was two orders of magnitude smaller than the delta-T flicker noise.

SECTION 2: EXPERIMENT

V. SAMPLE PREPARATION

Molecular junctions were prepared in a mechanically-controllable break junction setup located within a cryogenic chamber, as described in Refs. 1 and 8. The chamber is first pumped to 10^{-5} mbar and then cooled using liquid helium to ~ 4.2 K. Samples are made of a notched Au wire (99.99% purity, 0.1 mm diameter, 25 mm length, Goodfellow) that is attached to a flexible substrate (0.76 mm thick insulating Cirlex film). A three-point bending mechanism is used to break the wire at the notch (Fig. 1a) and expose two ultra-clean atomically-sharp tips in cryogenic vacuum that serve as the junction's electrodes. The breaking process is controlled by a piezoelectric element (PI P-882 PICMA), which is connected to a Piezomechanik SVR 150/1 piezo driver, and is driven by a 24-bit NI-PCI4461 data acquisition (DAQ) card. These components allow achieving fast control over the distance between the two tips with sub-angstrom resolution. To form molecular junctions, hydrogen (99.999% purity, Gas Technologies) was introduced from an external cylinder to the cold junction via a stainless steel capillary. During the admission process, the formation of Au/hydrogen junctions was monitored by recording deviations from the typical conductance of bare Au (see Fig. S2).

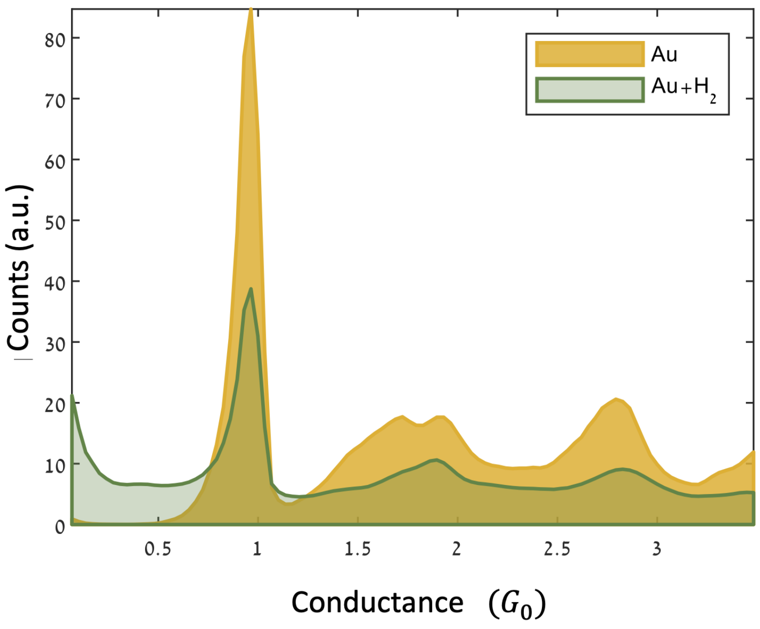

FIG. S2. **Most probable conductance of Au and Au/hydrogen junctions.** Conductance histograms of Au atomic junctions before (yellow) and after (green) the introduction of hydrogen to the junction. Each histogram is composed from at least 1,500 measurements of conductance as a function of electrode displacement conducted on different junctions at an applied voltage of 100 mV.

VI. CONDUCTANCE MEASUREMENTS

To measure the conductance of atomic and molecular junctions, we probed direct-current (d.c.) versus applied voltage while keeping inter-electrode distance constant. The conductance was extracted from the current-voltage curve by dividing the current by the voltage in the linear regime (±4 mV, in our case). The voltage was applied from a NI-PCI4461 DAQ, and the generated current was amplified by a current preamplifier (SR570) and recorded by the same DAQ card. Following each junction analysis, the two electrodes were squeezed against each other up to a conductance of at least 50 G_0 to ensure that the data consists of a statistical variety of different atomic-scale junction geometries. To minimize unwanted noise, the mentioned instruments, and the break junction system were placed in a Faraday cage and connected to a quiet ground. These instruments were optically isolated from a control computer located outside the Faraday cage. Batteries were used as a power source for the amplifiers to avoid noise injection from power lines. To further reduce extrinsic unwanted noise, including mechanical noise from the piezoelectric element, we connected an RC filter (R-resistance, C-capacitance) between the piezo driver and the piezoelectric

element. Before and after each noise measurement, a current versus voltage measurement was taken to find the conductance of the junction. The two measurements were compared to verify that the junction was intact during noise measurements.

To characterize the most probable conductance of Au and Au/hydrogen junctions, direct-current (d.c.) was measured while the junction was gradually broken by increasing the voltage applied to the piezoelectric element at a constant speed of 600 nm s^{-1} and a sampling rate of 100 kHz. The resulted current was divided by the applied voltage to give the conductance during junction elongation. As mentioned, after each conductance versus elongation measurement, the exposed atomic tips were pushed back into contact until the conductance reached a value of at least 50 G_0 to sample atomic-scale junctions with different geometries. The conductance histograms in Fig. S2 were constructed based on these measurements.

VII. CHARACTERIZATION OF AU AND AU/HYDROGEN JUNCTIONS

The most probable conductance of Au single-atom junctions is ~ 1 G_0, dominated by a single transmission channel[9–11]. Stretching Au atomic junctions can reduce the conductance typically down to 0.8 G_0, and in rare cases down to 0.75 G_0. To be able to study noise characteristics of junctions with conductance below this value, hydrogen was introduced to create stable molecular junctions with a wider conductance range[1,8] below 1 G_0. Before the admission of molecules, the bare Au junctions were characterized by constructing conductance histograms, as seen in Fig. S2 (yellow). The main peak at 1 G_0 and the tail at low conductance are known as the typical signature of a bare Au atomic junction[12,13]. This peak provides the most probable conductance of a single atom Au junction, and the low conductance tail is the consequence of tunneling conductance detected after breaking a single atom junction. Following the introduction of hydrogen, the conductance histogram reveals different characteristics as found in Fig. S2 (green). The large number of counts below 1 G_0 indicates the repeated formation of a variety of stable molecular junction geometries with a wide range of conductance values. This characteristics allows us to conduct noise measurements on stable junction geometries with a broad range of conductance values below 1 G_0.

VIII. TEMPERATURE MEASUREMENTS

A silicon diode thermometer was attached to each electrode near the electrode tips (Fig. 1a). The probing electric wires from the thermometers were attached to metal thermalization plates characterized with a temperature of ~ 4.2 K to reduce absorption of heat from the hot side of the wires, outside the cryostat at ~ 300 K. As a result, when the junction is heated above the base temperature, the probed temperature by the thermometers is always lower than the junction's temperature, as indicated by thermal noise (Nyquist-Johnson noise) measurements. However, with the aid of thermal noise measurements, we could calibrate the temperature indicated by the thermometer to give the temperature in the nanoscale vicinity of the studied junction. Note that thermal noise identifies the electronic temperature that is defined by the Fermi–Dirac distribution of electrons within the electrodes, typically in a region of tens to hundreds of nanometers around the atomic scale junction at 4.2 K. Thermal noise as a function of conductance was measured at several fixed temperatures. Then, the relation between the temperature given by the thermometers and the temperatures given by thermal noise was found for the relevant temperature range in our experiment. Before each experiment, the mentioned calibration procedure was used to relate a temperature at the nanoscale vicinity of the junction to the thermometer reads. Additional relevant information can be found in Ref. 8.

IX. MEASUREMENTS OF FLICKER NOISE AT FINITE TEMPERATURE DIFFERENCES

After the formation of an atomic scale junction with a fixed inter-electrode distance at a given temperature difference, a current as a function of voltage curve was measured and the conductance was determined from the curve's slope ($G = I/V$) at its linear regime around zero voltage. The junction's voltage noise was amplified ($\times 10^5$) by a specially-made differential low-noise voltage amplifier and analyzed using a NI PXI-5922 DAQ card with the aid of a LabView implemented fast Fourier transform (FFT) analysis. The noise at a given temperature difference was probed as a function of frequency in a range of 0.25-300 kHz and averaged 1,000 times. To ensure the junction's stability during this procedure, a second current-voltage measurement was done after the noise measurement. Only when the difference between the measured conductance values before and after the noise measurement was lower than ~ 1%, we considered the noise measurement to be pertinent for this study.

The unwanted voltage noise contribution of the setup output was measured for a shorted (mechanically squeezed) junction at the same temperature difference and was subtracted from the total noise spectra found in the experiment. For our setup, this voltage noise was typically 0.90-0.95 nV/$Hz^{1/2}$. The remaining noise spectra was subjected to low-pass RC filtering, as a result of the finite setup's resistance and capacitance. Moreover, this noise contained a finite contribution from the amplifier input current noise that was also suppressed by RC filtering. To account for these effects, thousands of noise as a function of frequency spectra were measured at different conductance and temperature (at zero temperature difference) in the relevant range of our analysis (0.1-7.0 G_0 and 5.4-50.4 K). The capacitance was determined by fitting an RC function (in units of

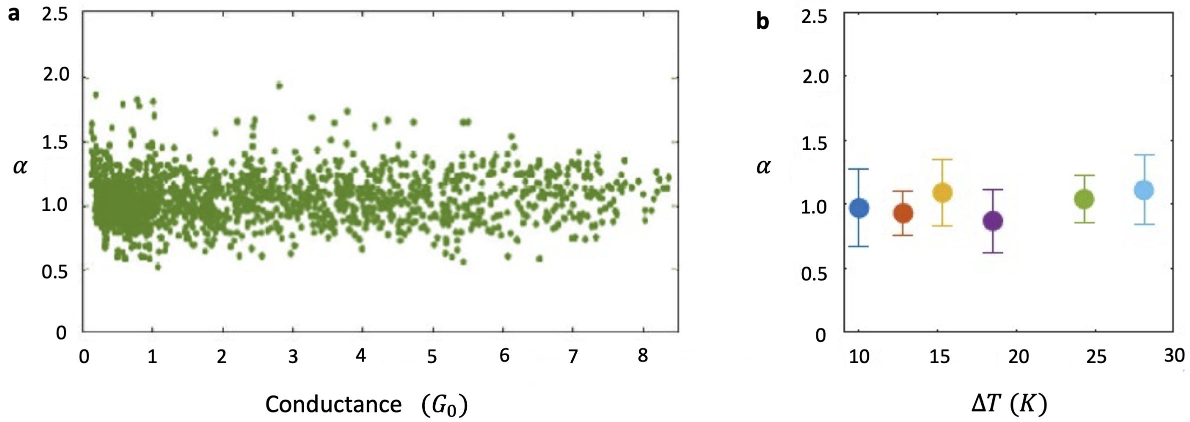

FIG. S3. **Frequency dependence of delta-T flicker noise measured in Au/hydrogen junctions. a,** Extracted α by fitting the Hooge's expression[12]: $S_f \sim 1/f^{\alpha}$ to the measured ΔT flicker noise at an average temperature of 25.7±0.6 K and a temperature difference of 24.3±0.5 K. **b,** Average α for different temperature differences and an average temperature presented in Fig. 3b from left to right, respectively. α is scattered around 1 for ΔT flicker noise, as in the case of flicker noise detected by an applied voltage[1]. Error bars represent the α standard deviation.

V^2/Hz): $S = S_0/[1 + (2\pi f RC)^2]$, where S_0 is the zero frequency total noise. The amplifier input current noise was extracted by: $S_0 = 4k_BTR + [S_I^{in}(f)]^2R^2$ (S_0 is in units of V^2/Hz). We found a typical capacitance of $C = 42.4 \pm 0.1$ pF for our measurement system and an amplifier input current noise of: $S_I^{in}(f) = 1.37 \times 10^{-32} f$.

Once the capacitance and amplifier input current noise were found, every total noise spectrum that was measured at a finite temperature difference was corrected by the inverse of the RC function, using the obtained resistance from the above described conductance measurements ($R = 1/G$). The determined amplifier input current noise was subtracted from the total noise to have the corrected total noise.

Next, the white noise contribution (essentially, thermal noise and delta-T white noise) was determined at the frequency range of 280-290 kHz, for which flicker noise is negligible. This contribution was subtracted from the corrected total noise. The resulted excess noise (Fig. 1c) represents the delta-T flicker noise contribution. This voltage noise was converted to current noise data (with units of A^2/Hz) by dividing each value by the square of the corresponding resistance, R2. The obtained delta-T flicker noise was fitted to Hooge's expression[14]: $S_f \sim 1/f^{\alpha}$ to find $\alpha \approx 1$, as demonstrated in Fig. S3. Finally, the excess noise was integrated in the range of 1-10 kHz, in order to study the delta-T flicker noise as a function of conductance in view of equation (2).

X. MEASUREMENTS OF FLICKER NOISE AT A FINITE CURRENT BIAS

To measure voltage-bias flicker noise in atomic and molecular junctions, the studied junctions were current-biased by a Yokogawa GS200 SC voltage source connected to the sample via two 0.5 M or 1 M resistors placed near the junction. The rest of the measurement and analysis procedure were carried out similarly to the above-described measurements of flicker noise at finite temperature differences. The resulting excess noise[15] represented the voltage-bias flicker noise component. This excess noise was integrated in the range of 1-10 kHz, providing the flicker noise versus conductance data that was utilized to determine the values of S_{min} and S_{max} as seen in Fig. S4. The two prefactors were necessary in order to identify the range of expected voltage-bias flicker noise due to the presence of thermovoltage (Fig. 2 in pink).

XI. THERMOVOLTAGE MEASUREMENTS

The thermovoltage of the system was measured at the temperature difference considered in Fig. 2a (15.1±0.3 K). The measurement procedure is based on the technique described in Ref. 16. In Fig. 2b we present a histogram of the measured total thermovoltage Au/hydrogen junctions. The scattering of the thermovoltage can be escribed to structural variations between the examined atomic scale junctions.

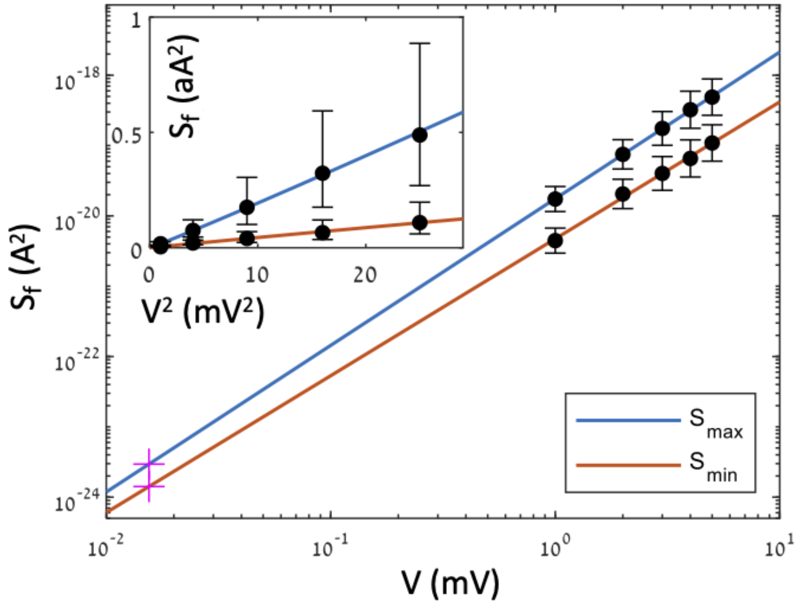

FIG. S4. **Flicker noise prefactor extracted at different applied voltage.** S_{min} and S_{max} are the minimal and maximal prefactors found by fitting Eq. (1) of the main text to voltage flicker noise in Au/hydrogen junctions in a range of 0.1-1.0 G_0, assuming a single transmission channel. The procedure is described in detail in Ref.[1]. The blue and red fits allow finding S_{min} and S_{max} relevant for the voltage flicker noise produced by thermoelectric voltage 18 μV. Pink marks indicate the prefactors at a voltage equal to the thermoelectric voltage. Inset: Similar presentation in a linear scale. Error bars represent the log standard deviation.

---

[1] O. Shein-Lumbroso, J. Liu, A. Shastry, D. Segal, and O. Tal, Quantum Flicker Noise in Atomic and Molecular Junctions, Phys. Rev. Lett. **128**, 237701 (2022).

[2] B. Ludolph and J.M. van Ruitenbeek, Conductance fluctuations as a tool for investigating the quantum modes in atomic-size metallic contacts, Phys. Rev. B **61**, 2273 (2000).

[3] F. Liefrink, J.I. Dijkhuis, and H. van Houten, Low frequency noise in quantum point contacts, Semicond. Sci. Technol. **9**:2178 (1994).

[4] G. Margolin and E. Barkai, Nonergodicity of a Time Series Obeying Lévy Statistics, J. Stat. Phys. **122**, 137 (2006).

[5] S. Engelberg, *A Mathematical Introduction to Control Theory*, (Imperial College Press, U.K., 2005).

[6] C.W.J. Beenakker, Random-matrix theory of quantum transport, Rev. Mod. Phys. **69**, 731 (1997).

[7] C.W.J. Beenakker and M. Büttiker, Suppression of shot noise in metallic diffusive conductors, Phys. Rev. B **46**, 1889(R) (1992).

[8] O.S. Lumbroso, L. Simine, A. Nitzan, D. Segal, and O. Tal, Electronic noise due to temperature differences in atomic-scale junctions, Nature **562**, 240-244 (2018).

[9] H. E. Van den Brom, and J. M. Van Ruitenbeek, Quantum suppression of shot noise in atom-size metallic contacts. Phys. Rev. Lett. **82**, 1526 (1999).

[10] M. Dreher, F. Pauly, J. Heurich, J. C. Cuevas, E. Scheer, and P. Nielaba, Structure and conductance histogram of atomic-sized Au contacts, Phys. Rev. B **72**, 075435 (2005).

[11] F. Pauly, J. K. Viljas, M. Bürkle, M. Dreher, P. Nielaba, and J. C. Cuevas, Berry phase and pseudospin winding number in bilayer graphene, Phys. Rev. B **84**, 195420 (2011).

[12] B. Ludoph, M. H. Devoret, D. Esteve, C. Urbina, and J. M. Van Ruitenbeek, Evidence for saturation of channel transmission from conductance fuctuations in atomic-size point contacts, Phys. Rev. Lett. **82**, 1530 (1999).

[13] G. Rubio-Bollinger, C. de Las Heras, E. Bascones, N. Agraït, F. Guinea, and S. Vieira, Single-channel transmission in gold one-atom contacts and chains, Phys. Rev. B **67**, 121407 (2003).

[14] M. B. Weissman, $1/f$ noise and other slow, nonexponential kinetics in condensed matter, Rev. Mod. Phys. **60**, 537 (1988).

[15] A. L. Goldberger, L. A. Amaral, J. M. Hausdorff, P. C. Ivanov, C. K. Peng, and H. E. Stanley, Fractal dynamics in physiology: alterations with disease and aging, Proc. Natl. Acad. Sci. **99**, 2466-2472. (2002).

[16] C. Evangeli, K. Gillemot, E. Leary, M. T. Gonzalez, G. Rubio-Bollinger, C. J. Lambert, and N. Agrait, Engineering the thermopower of C60 molecular junctions, Nano Lett. **13**, 2141-2145 (2013).
